# Cost of Routine Herpes Simplex Virus Infection Visits to U.S. Emergency Departments 2006–2013

**DOI:** 10.5811/westjem.2018.3.37543

**Published:** 2018-06-04

**Authors:** Fan Di Xia, Mary Fuhlbrigge, Erica Dommasch, Cara Joyce, Arash Mostaghimi

**Affiliations:** * Harvard Medical School, Brigham & Women’s Hospital, Boston, Massachusetts; † Brigham & Women’s Hospital, Boston, Massachusetts; ‡ Harvard Medical School, Beth Israel Deaconess Medical Center, Boston, Massachusetts; § Loyola University, Chicago, Illinois; # Harvard Medical School, Brigham & Women’s Hospital, Department of Dermatology, Boston, Massachusetts

## Abstract

**Introduction:**

Little is known about emergency department (ED) utilization for herpes simplex viruses (HSV) types 1 and 2 in the United States. Our goal was to determine the utilization and cost burden associated with HSV infection visits to U.S. EDs in recent years from 2006–2013.

**Methods:**

We analyzed the Nationwide Emergency Department Sample (NEDS) database, the largest national database of hospital-based ED visits in the U.S., to determine the number of visits and the cost associated with HSV visits from 2006–2013. We also analyzed trends across years.

**Results:**

From 2006–2013, there were 704,728 ED visits with a primary diagnosis of HSV infection. Of these, 658,805 (93.5%) resulted in routine discharges without inpatient admission, amounting to a total ED charge of $543.0 million. After adjusting for inflation, there was a doubling of total ED spending for HSV from 2006 to 2013 ($45.0 million to $90.7 million) and a 24% increase in number of visits (73,227 visits in 2006, vs. 90,627 visits in 2013). ED visits for genital herpes have increased while visits for herpes gingivostomatitis have decreased.

**Conclusion:**

HSV-associated ED use and associated costs have increased between 2006–2013. Most of these cases could likely be managed in non-emergent outpatient settings as 93.5% of visits resulted in routine discharges without admission. Our findings add to knowledge regarding HSV utilization and epidemiology in the U.S. and highlight the need for continued prevention, patient education, and emphasis of care in non-emergency settings to prevent unnecessary ED utilization.

## INTRODUCTION

Herpes simplex virus type 1 (HSV-1) and type 2 (HSV-2) are common viral infections with an estimated seroprevalence of 53.9% and 15.7% in the United States, respectively, from 2005–2010.^1^ Although both viruses can have systemic sequelae, uncomplicated HSV infections are most commonly self-limited and treated in an outpatient non-emergent setting. Despite the commonality of HSV, little is known about the incidence of symptomatic cases and the economic burden of HSV infection on national healthcare expenditures. In this study, we aimed to characterize the utilization and cost burden associated with HSV infection visits to U.S. emergency departments (ED) from 2006 through 2013.

## METHODS

We used the Healthcare Cost and Utilization Project Nationwide Emergency Department Sample (NEDS) database, which is the largest all-payer national database of hospital-based ED visits in the U.S.^2^ The database contains data for roughly 30 million ED visits each year and approximately 135 million weighted ED visits in total. The database contains information such as diagnoses of ED visits searchable through *International Classification of Diseases, Ninth Revision, Clinical Modification* (ICD-9-CM) codes, patient demographic information, ED charges for ED visits, and total hospital charges for ED-related admissions.^2^ We searched the database for visits for HSV infection in the years 2006–2013 using ICD-9-CM codes 054.0–054.9. These codes have a positive predictive value of 86% in identifying cases of HSV infection.^3^

We identified HSV ED visits with routine discharges – defined as visits that did not result in an in-patient admission – in the NEDS database, and we calculated visit counts and total ED charges using demographic and clinical variables. Annual charges were adjusted for inflation to 2016 dollars using the Medical Care Consumer Price Index.^5^ A multivariable linear regression model was constructed to calculate adjusted mean charges. We used survey procedures in SAS 9.4 to produce national estimates based on the stratified, single-stage cluster design of the NEDS database. This study was deemed exempt by the Partners Healthcare institutional review board.

## RESULTS

From 2006–2013, a total of 1,024,771,257 visits were made to the ED, 704,728 (0.069%) of which were visits with a primary diagnosis of HSV infection. Of these, 658,805 (93.5%) were ED visits with routine discharges that did not result in an inpatient admission, amounting to a total ED charge of $543.0 million**.** The mean age of patients was 25.2 years, 63.6% of whom were female (Table 1).

Adjusted mean visit charges were higher for those age ≥ 50 years (p=.010), female (p<.001), diagnosed with genital herpes or herpes simplex with complication (p<.001), with concurrent chronic conditions (p<.001), or with private insurance (p<.001) (Table 1).

Total HSV ED visits and spending, after adjusting for inflation, increased annually from 2006–2013 (73,227 visits, $45.0 million in 2006 vs. 90,627 visits, $90.7 million in 2013). Annual visits for genital herpes have increased (n=24,747, 33.8% in 2006 vs. n=36,518, 40.3% in 2013) while visits for herpetic gingivostomatitis decreased (n=14,934, 20.4% in 2006 vs. n=12,061, 13.3% in 2013) (Table 2).

## DISCUSSION

Our study demonstrates that routine HSV infection accounted for 658,805 ED visits and $543.0 million in ED charges over eight years from 2006–2013. Trends across years, after adjusting for inflation, show an approximate doubling of ED spending on routine HSV infections from 2006 to 2013 ($45.0 million to $90.7 million), and a 24% increase in number of visits (73,227 visits in 2006, vs. 90,627 visits in 2013).

ED visits for genital herpes between 2006 and 2013 have increased while visits for herpes gingivostomatitis have decreased. This finding is consistent with reports of increased rates of genital HSV-1 infections in the setting of possible lack of protection from pre-existing orolabial HSV-1 antibodies, and it highlights the evolving epidemiology of this disease. 1

These findings add to the existing literature on HSV prevalence and epidemiology in the U.S. by providing data on the national ED utilization and charge pattern for HSV infection. Routine HSV infections can largely be treated in non-urgent, outpatient settings. In our cohort, 68% of the patients had insurance coverage, while only 3.9% had herpes simplex with complication, suggesting that the majority of ED utilization for HSV infection could have been transitioned to non-urgent care settings to prevent unnecessary ED use.

Adjusted mean ED charges were higher for females ($981, p<.001) or those with private insurance ($943, p<.001). Protocol differences between ED management of males vs. females (e.g., routine human chorionic gonadotropin urine tests for females) may have contributed to the differences; additionally, higher costs may also be charged to private insurances as compared to Medicare and Medicaid. The overall increase in cost could be a function of increased diagnostic evaluation such as direct fluorescent-antibody testing. Unfortunately, this dataset does not provide itemized charges, so direct contributors to cost cannot be determined.

Limited access to primary care, convenience of ED access, and patient alarm in the case of genital herpes may have played a role in the utilization of the ED for routine HSV infections. Younger patients are more likely to visit the ED for non-urgent conditions and may be a target for future intervention.^6^ Public health efforts should focus on patient education and improving alternative access to care to reduce reliance on ED services for HSV. Efforts to provide easier access to medications via teledermatology consultation or over-the-counter access, especially for patients with established diagnoses may reduce utilization.

## LIMITATIONS

Our findings should be interpreted in the context of the study design. The NEDS database provides ED/hospital charges but does not have information regarding reimbursed amounts or fees paid to physicians and other professionals.^2^ As with other NEDS studies, charges may not be fully reimbursed, and thus our findings in this study may overestimate the overall costs. This limitation is unlikely to significantly impact the year-to-year comparison and overall trends.

## CONCLUSION

In summary, our study demonstrates the increasing costs associated with treatment of HSV in U.S. EDs. As most of these patients were routine discharges, much of this care could have likely been provided in alternative, lower-cost settings. Our findings highlight the need for continued prevention, patient education, and emphasis of care in non-emergency settings to prevent unnecessary ED use for routine HSV infections.

## Figures and Tables

**Table 1:** Nationwide herpes simplex virus infection emergency department routine disposition visits and costs 2006–2013.

	Visits with primary diagnosis of herpes simplex, n (%)	Total ED charge amount, $	Adjusted mean ED charge amount, $ (SE)	P value
Overall	658805	543042020		
Age				
< 30	460176 (69.9)	377448792	893 (22)	0.010
30–49	147017 (22.3)	123345760	865 (23)	
≥ 50	51612 (7.8)	42247468	904 (27)	
Gender				
Male	239536 (36.4)	164701372	793 (22)	<0.001
Female	419168 (63.6)	378291212	981 (23)	
Month of visit				
December – February	133962 (24.5)	102712319	893 (23)	0.007
March – May	136142 (24.9)	101231443	868 (23)	
June – August	141704 (25.9)	108318381	886 (23)	
September – November	135313 (24.7)	105544641	902 (22)	
Primary diagnosis				
Genital herpes	245484 (37.3)	278335295	1069 (21)	<0.001
Herpetic gingivostomatitis	115726 (17.6)	71019956	773 (18)	
Herpetic whitlow	19976 (3.0)	13124601	771 (21)	
Herpes simplex with complication	25717 (3.9)	22295131	1079 (84)	
Herpes simplex without mention of complication	251903 (38.2)	158267035	743 (15)	
Chronic Condition Indicator				
Concurrent chronic condition present*	339708 (51.6)	338918844	986 (24)	<0.001
No concurrent chronic condition	319098 (48.4)	204123176	788 (22)	
Primary payer				
Medicare	32993 (5.0)	64007415	905 (29)	<0.001
Medicaid	233494 (35.6)	103960789	859 (22)	
Private insurance	179528 (27.4)	112467580	943 (25)	
Self-pay	177287 (27.0)	67474745	868 (22)	
Other	32985 (5.0)	12990891	861 (38)	

*SE*, standard error; *ED*, emergency department.

* The NEDS database defines a chronic condition as “a condition that lasts 12 months or longer and meets one or both of the following tests: (a) it places limitations on self-care, independent living, and social interactions; (b) it results in the need for ingoing intervention with medical products, services, and special equipment (see Perrin et al., 1993). The identification of chronic conditions is based on all 5-digit ICD-9-CM codes. E Codes, or external injury codes, are not classified, because all injuries are assumed to be acute.” Subcategories may not sum to totals due to missing values.

**Table 2:** Nationwide herpes simplex emergency department (ED) visit and charges 2006–2013 by year.

Year, n	2006	2007	2008	2009	2010	2011	2012	2013
No. of visits, n	73227	77477	76227	78001	88063	86456	88729	90627
Age, n (%)								
< 30	51200 (69.9)	54599 (70.5)	54313 (71.3)	55495 (71.1)	61427 (69.8)	60395 (69.9)	61074 (68.8)	61671 (68.0)
30–49	16590 (22.7)	16945 (21.9)	16357 (21.5)	16642 (21.3)	19862 (22.6)	19086 (22.1)	20308 (22.9)	21227 (23.4)
≥ 50	5436 (7.4)	5932 (7.7)	5557 (7.3)	5863 (7.5)	6773 (7.7)	6975 (8.1)	7346 (8.3)	7729 (8.5)
Gender, n (%)								
Male	27736 (37.9)	28431 (36.7)	27927 (36.6)	28602 (36.7)	31353 (35.6)	31155 (36.0)	32163 (36.3)	32168 (35.5)
Female	45486 (62.1)	49030 (63.3)	48299 (63.4)	49343 (63.3)	56704 (64.4)	55301 (64.0)	56543 (63.7)	58459 (64.5)
Primary diagnosis, n (%)								
Genital herpes	24747 (33.8)	26440 (34.1)	27484 (36.1)	28440 (36.5)	33258 (37.8)	33095 (38.3)	35501 (40.0)	36518 (40.3)
Herpetic gingivostomatitis	14934 (20.4)	15620 (20.2)	14802 (19.4)	14154 (18.1)	14908 (16.9)	15691 (18.1)	13557 (15.3)	12061 (13.3)
Herpetic whitlow	2227 (3.0)	2305 (3.0)	2376 (3.1)	2144 (2.7)	2589 (2.9)	2606 (3.0)	2796 (3.2)	2934 (3.2)
Herpes simplex w/complication	3762 (5.1)	2775 (3.6)	2047 (2.7)	2649 (3.4)	3413 (3.9)	3492 (4.0)	3455 (3.9)	4123 (4.5)
Herpes simplex w/o complication	27557 (37.6)	30336 (39.2)	29517 (38.7)	30615 (39.2)	33894 (38.5)	31572 (36.5)	33421 (37.7)	34991 (38.6)
Total ED charge amount, $	44973742	51725820	55437779	61336526	76707637	78232385	83950203	90677926
